# Oligoclonal immunoglobulin repertoire in biliary remnants of biliary atresia

**DOI:** 10.1038/s41598-019-41148-7

**Published:** 2019-03-14

**Authors:** Sarah A. Taylor, Padmini Malladi, Xiaomin Pan, Joshua B. Wechsler, Kathryn E. Hulse, Harris Perlman, Peter F. Whitington

**Affiliations:** 1 Department of Pediatrics, Ann and Robert H Lurie Children’s Hospital of Chicago, Chicago, Illinois United States; 2 Stanley Manne Children’s Research Institute, Chicago, Illinois United States; 3 Department of Medicine, Northwestern University, Chicago, Illinois United States

## Abstract

Biliary atresia (BA) is a neonatal cholestatic liver disease that is the leading cause of pediatric liver transplantation, however, the mechanism of disease remains unknown. There are two major forms of BA: isolated BA (iBA) comprises the majority of cases and is thought to result from an aberrant immune response to an environmental trigger, whereas syndromic BA (BASM) has associated malformations and is thought to arise from a congenital insult. To determine whether B cells in BA biliary remnants are antigen driven, we examined the immunoglobulin (Ig) repertoire of diseased tissue from each BA group. Deep sequencing of the Ig chain DNA was performed on iBA and BASM biliary remnants and lymph nodes obtained from the Childhood Liver Disease Research Network (ChiLDReN) repository. Statistical analysis of the Ig repertoire provided measures of Ig clonality and the Ig phenotype. Our data demonstrate that B cells infiltrate diseased iBA and BASM biliary remnant tissue. The Ig repertoires of iBA and BASM disease groups were oligoclonal supporting a role for an antigen-driven immune response in both sub-types. These findings shift the current understanding of BA and suggest a role for antigen stimulation in early iBA and BASM disease pathogenesis.

## Introduction

Biliary atresia (BA) is a progressive obliterative cholangiopathy of infancy, which often leads to end-stage liver disease and the need for transplantation in the first two years of life. In general, there are two major types of BA. Isolated biliary atresia (iBA) is most common and is defined as BA alone, with no other anomalies. The less common type is referred to as syndromic BA, or BA with splenic malformation syndrome (BASM), wherein congenital malformations including laterality defects accompany liver disease^1^. iBA and BASM have been thought to be fundamentally different in pathogenesis although the biliary pathology of both is characterized by fibro-obliteration of the extra-hepatic bile duct. BASM is hypothesized to arise from a congenital insult, whereas iBA is thought to result from a post-natal trigger leading to an aberrant immune response that causes destruction of the extra-hepatic bile ducts^2^. However, clinical observation of elevated conjugated bilirubin levels in infants with iBA within the first 48 hours of life suggest that the onset of BA may be earlier than previously thought^3^. While evidence supports the premise that multiple host factors contribute to BA^4^, we focus our current study on the B cell immune response to advance the understanding of BA with the ultimate goal to develop improved diagnostic and treatment strategies.

While the precise etiology of BA remains unknown, T cell immunity has been implicated in disease pathogenesis^5^. An oligoclonal T cell receptor repertoire in diseased human BA liver and bile duct remnant samples supports the hypothesis that antigen stimulation is involved early in the disease course of BA^6^. While prior work has suggested B cells are also involved in BA, it remains unclear if their primary function in disease pathogenesis is antibody production, antigen presentation, or cytokine-mediated regulation of other immune cells including T cells. Immunoglobulin deposits have been demonstrated in bile duct remnants in 34% of cases of human BA at the time of Kasai portoenterostomy^7^. In addition, research using the Rhesus-rotavirus (RRV)-induced mouse model of BA revealed that B cell deficient mice fail to develop biliary obstruction and have decreased Th1 cell activation^8^. Treatment with intravenous immunoglobulin in this murine model also decreased Th1 inflammation and increased the rate of extrahepatic bile duct patency although overall survival remained unchanged^9^. More recently, cytokine-mediated immune activation by neonatal B cells was implicated in the pathogenesis of murine BA rather than an antigen-dependent mechanism^10^.

Increasing work on the immunoglobulin (Ig) repertoire in specific disease states has provided insight into the role that B cell immunity plays in pathogenesis^11^. Individual B cells display a B cell receptor (BCR) that is equivalent to the Ig (or antibody) that the B cell produces, which is encoded by the RNA of the cell. The variable region of Ig is responsible for binding a specific antigen and consists of a unique combination of heavy (V, D, and J) and light (V and J) chain variable gene segments^12^. Recombination of these variable gene segments is the primary mechanism by which each B cell generates a unique Ig sequence. Random recombination provides the circulating B cell population in adult humans with 3 to 9 million unique heavy chain complementarity-determining region 3 (CDR3) sequences, the sequence within the variable region most closely associated with antigen specificity^13^. An individual’s Ig repertoire is further refined by exposure to specific antigens. Upon antigen stimulation, somatic hypermutation of variable region genes allows for additional variable gene sequence diversity and selection of high-affinity, antigen-specific Igs that provide a focused disease-specific antibody repertoire.

In the present study, we define the Ig repertoire of BA to determine if it has features of an antigen-driven immune response. We hypothesized that BASM cases would display more polyclonal Ig repertoires in line with the prevailing hypothesis that BASM arises from a congenital insult. Contrary to our hypothesis, BCR sequencing of the Ig repertoires of both iBA and BASM are oligoclonal, thereby supporting a role for antigen stimulation in disease pathogenesis of *both* BA phenotypes.

## Results

### Clinical characteristics and patient demographics

Laboratory data obtained closest to the time of sample collection for biliary remnants and choledochal cyst (CC) cases is shown in Table 1. Consistent with the clinical findings seen in BA, all patients had elevated conjugated or direct bilirubin levels as well as elevated gamma-glutamyltransferase (GGT) levels (average 617 U/L, range 175–1108 U/L). Review of patient demographics demonstrated that 9 patients were of white race, 1 was black, and 2 of other race. Age at surgery for the four patients with CC ranged from <1 month of age to 30 months of age. At the time of sample collection, patients with CC were less cholestatic and had lower aminotransferase values than patients with BA.

**Table 1:** Serum laboratory data of patients obtained closest to tissue sample collection.

Sample	Age at labs	TB (mg/dL)	CB or DB (mg/dL)	ALT (U/L)	AST (U/L)	GGT (U/L)	Albumin (g/dL)
iBA1	1 mo	3.7	3.2 (CB)	79	101	781	3.5
iBA2	2 mo	7.3	4.2 (DB)	17	30	731	3.3
iBA3	2 mo	5.3	4.1 (CB)	216	353	1108	3.5
iBA4	1 mo	5.9	4.6 (CB)	547	207	467	2.5
iBA6	3 mo	5	3.4 (CB)	59	84	566	3.1
iBA8	1 mo	13.3	1.6 (DB)	15	51	964	2.8
iBA9	3 mo	8.1	6.5 (DB)	146	201	ND	4.1
iBA11	2 mo	7.4	ND	104	137	747	3.1
BASM12	2 mo	6.7	4.8 (CB)	200	254	563	4.4
BASM14	0 mo	8.3	6.5 (CB)	382	280	211	2.6
BASM15	2 mo	6	3.3 (CB)	148	219	479	4.6
BASM16	2 mo	9.6	5.4 (CB)	749	810	175	3.5
CC1	0 mo	2.8	1.3 (DB)	38	41	ND	2.0
CC2	30 mo	0.2	ND	16	39	ND	4.3
CC3	11 mo	0.2	<0.2 (DB)	17	44	ND	4.5
CC4	12 mo	1.2	0.3 (DB)	146	228	ND	3.7

Laboratory data was obtained the same week of tissue sample collection in all cases except for BASM 15 (sample collected 13 weeks after labs), BASM 16 (sample collected 4 weeks after labs), and CC3 (sample collected 8 weeks after labs). Age is reported rounded down to the nearest month. Abbreviations: mo, months; TB, total bilirubin; CB, conjugated bilirubin; DB, direct bilirubin; ALT, alanine aminotransferase; AST, aspartate aminotransferase; GGT, gamma-glutamyltransferase; ND, no data.

### B cell abundance in biliary remnants of infants with BA

**Figure 1:**
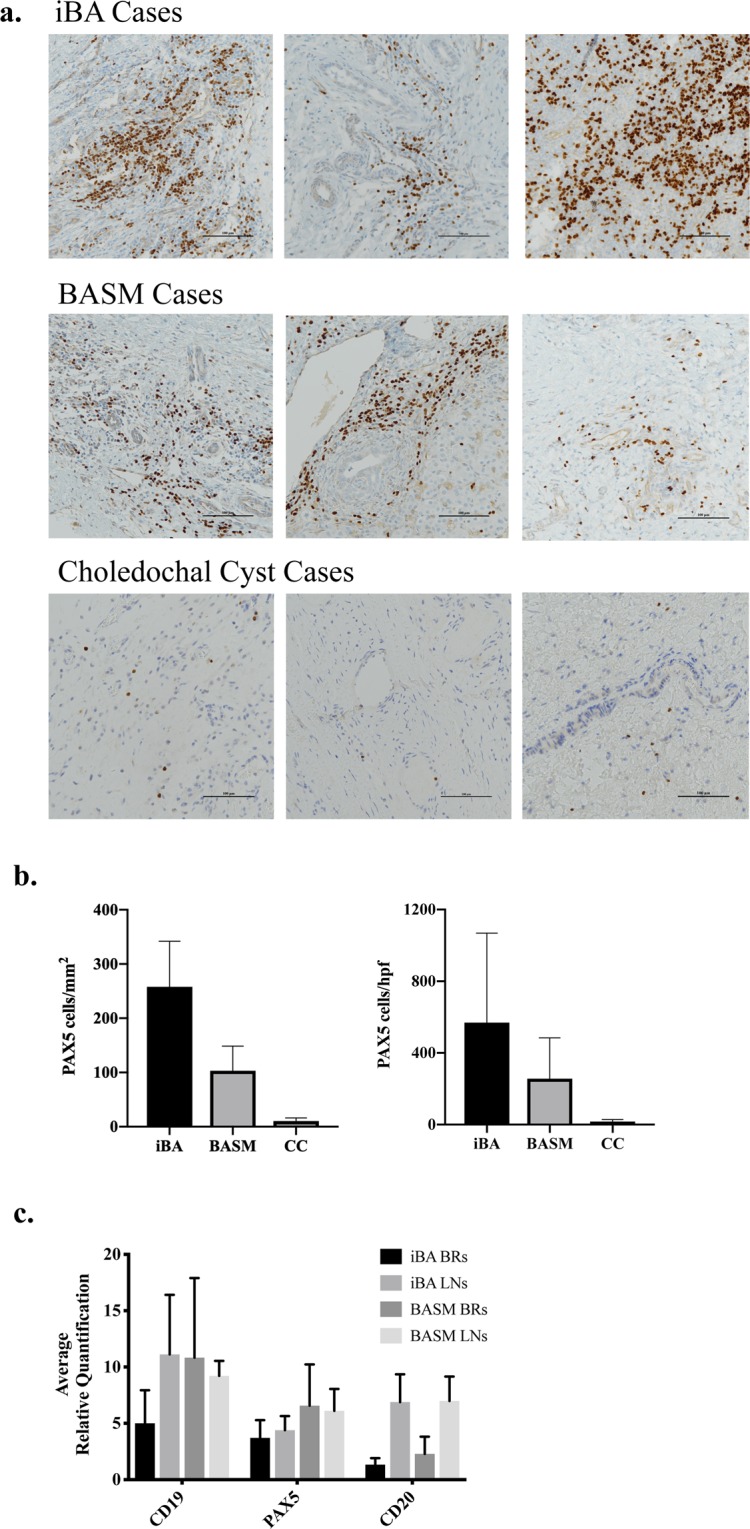
Abundant B cells are present in biliary remnants of iBA and BASM infants as compared to choledochal cyst cases. (**a**) High-powered field images demonstrating the densest areas of Pax5 positive B cells in 3 iBA, BASM, and CC cases. (**b**) Quantification of Pax5 positive B cells per mm^2^ of tissue (whole slide) and per the densest high-powered field (40x magnification) for iBA, BASM, and CC cases. (**c**) Average relative RT-PCR quantification of the B cell markers CD19, Pax5, and CD20 demonstrated no significant difference in expression levels between iBA and BASM biliary remnants (p > 0.05). There was no significant difference in relative quantification of CD19 and Pax5 between the remnants and their paired local lymph nodes (p > 0.05), however, CD20 expression was significantly lower in biliary remnants (p = 0.01).

Immunohistochemical staining for the B cell lineage marker Pax5 was performed to evaluate the abundance of B cells in biliary remnants from iBA and BASM cases. Though variable between cases, Pax5 positive B cells were present in samples from both iBA and BASM patients but very sparse in biliary tissue from CC cases (Fig. 1a). Quantification of B cells/mm^2^ and average B cells per high-powered field demonstrated that iBA cases had the greatest number of B cells (Fig. 1b). Both BA groups had many more B cells than CC cases although this was only statistically significant for full slide quantification of iBA v.s. CC due to variability between cases and low sample size (Fig. 1b). Unpaired t-test for comparison between number of B cells per mm^2^ and per high-powered field by group was 0.16 and 0.28 for iBA v.s. BASM, 0.026 and 0.062 for iBA v.s. CC, and 0.091 and 0.079 for BASM v.s. CC respectively.

To validate the immunohistochemistry studies we used RT-PCR to assess gene expression of the B cell markers CD19, Pax5, and CD20 in iBA and BASM biliary remnant tissue (Fig. 1b). Gene expression was compared between biliary remnants and paired lymph nodes to confirm the conditions, as well as provide additional evaluation of B cell numbers in biliary remnants, a non-immune tissue. Overall, relative quantification of these B cell markers in biliary remnant tissues was not significantly different between iBA and BASM biliary remnants. Average relative quantification for iBA versus BASM biliary remnants was 5.01 and 10.84 respectively for CD19 (p = 0.49), 3.71 and 6.57 respectively for Pax5 (p = 0.51), and 1.34 and 2.29 for CD20 (p = 0.59). Additionally, expression level of CD19 and Pax5 in the biliary remnants were not statistically different when compared to their paired lymph nodes. Average relative quantification for biliary remnants versus lymph nodes was 7.92 and 10.17 respectively for CD19 (p = 0.62), and 5.14 and 5.25 respectively for Pax5 (p = 0.96). However, average biliary remnant expression of CD20 was significantly lower than the paired lymph nodes. Average relative quantification for CD20 was 1.82 in biliary remnants as compared to 6.94 in the paired lymph nodes (p = 0.01). This difference may be due to greater abundance of antibody-secreting B cells in diseased biliary remnant tissue as compared to lymph nodes.

### Clonal expansion of the immunoglobulin repertoire in iBA and BASM biliary remnants

**Figure 2:**
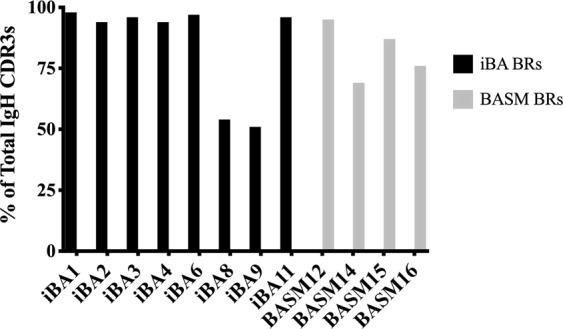
Clonality of iBA and BASM Ig repertoires. Analysis of the % contribution of the top 100 IgH CDR3 sequences demonstrates highly clonal Ig repertoires.

We next evaluated whether there was evidence for B cell clonal expansion in iBA and BASM. A summary of the sequencing data and clonality by sample is provided in Table 2. Evaluation of clonality by the D50 value demonstrated an average IgH D50 of 0.4 (range 0.2–0.9) for the iBA samples and 0.5 (range 0.6–1.0) for BASM. These values indicate a highly clonal population of B cells in the remnants of both iBA and BASM cases. In addition, the top 100 IgH CDR3s of both iBA and BASM biliary remnants comprised the majority of total CDR3 sequences, consistent with highly clonal Ig repertoires. In iBA the top 100 CDR3s comprised on average 85% (range 51–98%) and in BASM they comprised on average 82% (range 69–95%) of total CDR3s (Fig. 2).

**Table 2:** BCR sequencing data summary for biliary remnants and lymph nodes.

Biliary remnants	Reads	Distinct CDR3s	D50	Frequency top 100 CDR3s
iBA1	966609	2349	0.1	97.9%
iBA2	712470	6293	0.2	93.5%
iBA3	504036	3189	0.3	95.8%
iBA4	808102	4997	0.2	94.4%
iBA6	113811	1277	0.5	96.5%
iBA8	567690	12631	0.9	53.8%
iBA9	538696	12891	0.9	51.3%
iBA11	224469	2327	0.4	95.8%
BASM12	127299	2089	0.5	95.0%
BASM14	584857	9361	0.6	69.3%
BASM15	584219	7365	0.4	87.1%
BASM16	497679	7917	0.6	76.3%
**Lymph nodes**	**Reads**	**Distinct CDR3s**	**D50**	**Frequency top 100 CDR3s**
iBA LN6	443254	36954	23.0	5.7%
iBA LN7	369392	32088	20.2	6.8%
iBA LN9	339861	13263	1.8	37.0%
iBA LN10	343714	25247	12.2	17.7%
iBA LN11	856998	77852	16.3	9.1%
BASM LN12	422420	18560	4.0	22.8%
BASM LN13	423820	30205	11.9	15.3%
BASM LN14	366526	32962	15.0	10.5%
BASM LN15	378908	92206	8.0	15.0%
BASM LN16	458374	26120	8.4	16.4%

One biliary remnant (iBA5) was excluded due to insufficient tissue and three biliary remnants (iBA7, iBA10, BASM13) were excluded due to poor BCR sequencing quality. This yielded a total of 8 iBA biliary remnants, 4 BASM biliary remnants, 5 iBA lymph nodes (3 with paired biliary remnants), and 5 BASM lymph nodes (4 with paired biliary remnants).

Analysis of unique IMGT clonotypes among total submitted sequences within the iBA and BASM disease groups further demonstrated a restricted Ig repertoire with a small number of clonotypes. Total IgH IMGT clonotypes of the iBA disease group comprised 5.3% of total submitted sequences and 6.8% of total submitted sequences in the BASM disease group. Collectively, these analyses demonstrate that biliary remnants from both iBA and BASM contain highly clonal Ig repertoires.

### Ig phenotype of BA: CDR3 length distribution and variable gene use

**Figure 3:**
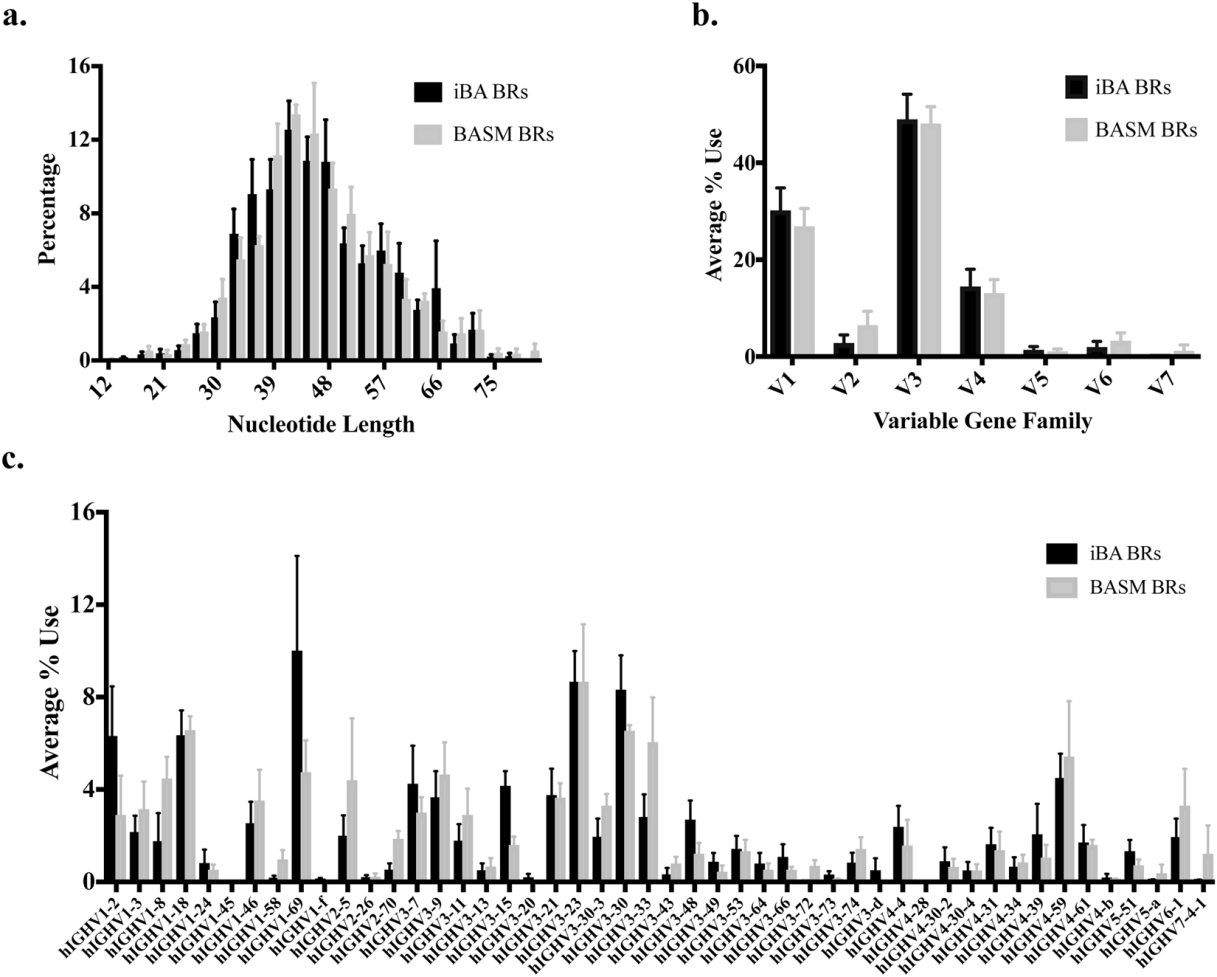
Immunoglobulin phenotype of BA. (**a**) CDR3 length distribution of iBA and BASM biliary remnants (BRs) demonstrated similar distribution with a dominant CDR3 length of 42 nucleotides. (**b**) Analysis of V-gene family usage demonstrated similar usage between iBA and BASM biliary remnant Ig repertoires. (**c**) Average use of V-gene alleles showed V1-69 was the most variable between iBA and BASM biliary remnants and V3-23 and V3-30 were two of the most commonly used alleles in both disease groups.

**Figure 4:**
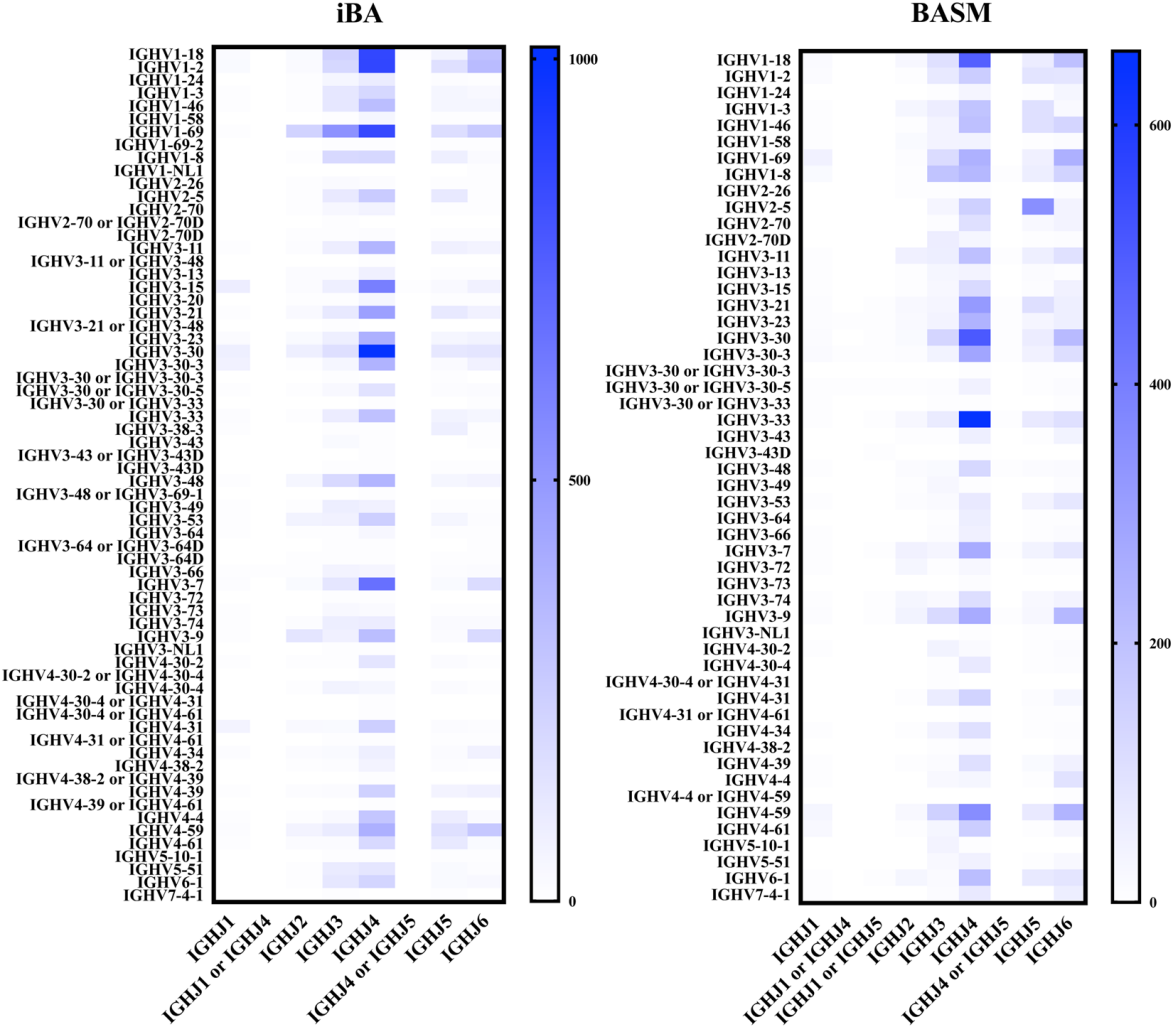
V-J gene associations by disease group. Heat map of V-J gene associations for iBA and BASM disease groups from IMGT/StatClonotype analysis shows that the disease groups share the common dominant associations V3-30/VJ-4 and V1-18/VJ-4.

To further characterize the disease-specific Ig repertoire of BA, we evaluated changes in the CDR3 length distribution and variable gene use. The Ig repertoire of iBA and BASM demonstrated a similar IgH CDR3 length distribution. Each group had a predominance of IgH CDR3 lengths of 39–48 nucleotides (13–16 amino acids) with an average of 43% of CDR3 sequences within this length in the iBA group and 46% in the BASM group (Fig. 3a). This contrasts with what one would observe in a diverse Ig repertoire in which the CDR3 length is expected to demonstrate a more truncated and discretized Gaussian distribution^14^. Variable gene family use within the heavy chain was also similar between iBA and BASM, with the V3 gene family being most dominant (Fig. 3b). Differences within the V-gene families were reflective of common allelic differences in the general population^15^. The V3-23 allele is commonly the most utilized IgH V-gene^15^ and as expected was one of the most dominant alleles in each disease group with an average usage of 9% in both the iBA and BASM Ig repertoires (Fig. 3c). We observed a high degree of variability in the use of the V1-69 allele between iBA and BASM consistent with known variability of this allele in the general population^15^. V1-69 was the most frequently used allele in the iBA group (10%) as compared to 5% in the BASM group (Fig. 3c). Analysis of the top V-J gene associations in IMGT/StatClonotype demonstrated similar associations between the iBA and BASM groups. Top V-J gene associations in iBA were V3-30/VJ-4, V1-2/VJ-4, V1-18/VJ-4, and V1-69/VJ-4 and in BASM were V3-33/VJ-4, V3-30/VJ-4, V1-18/VJ-4, and V4-59/J-4 (Fig. 4).

### Sequence variation in the BA immunoglobulin repertoire

**Figure 5:**
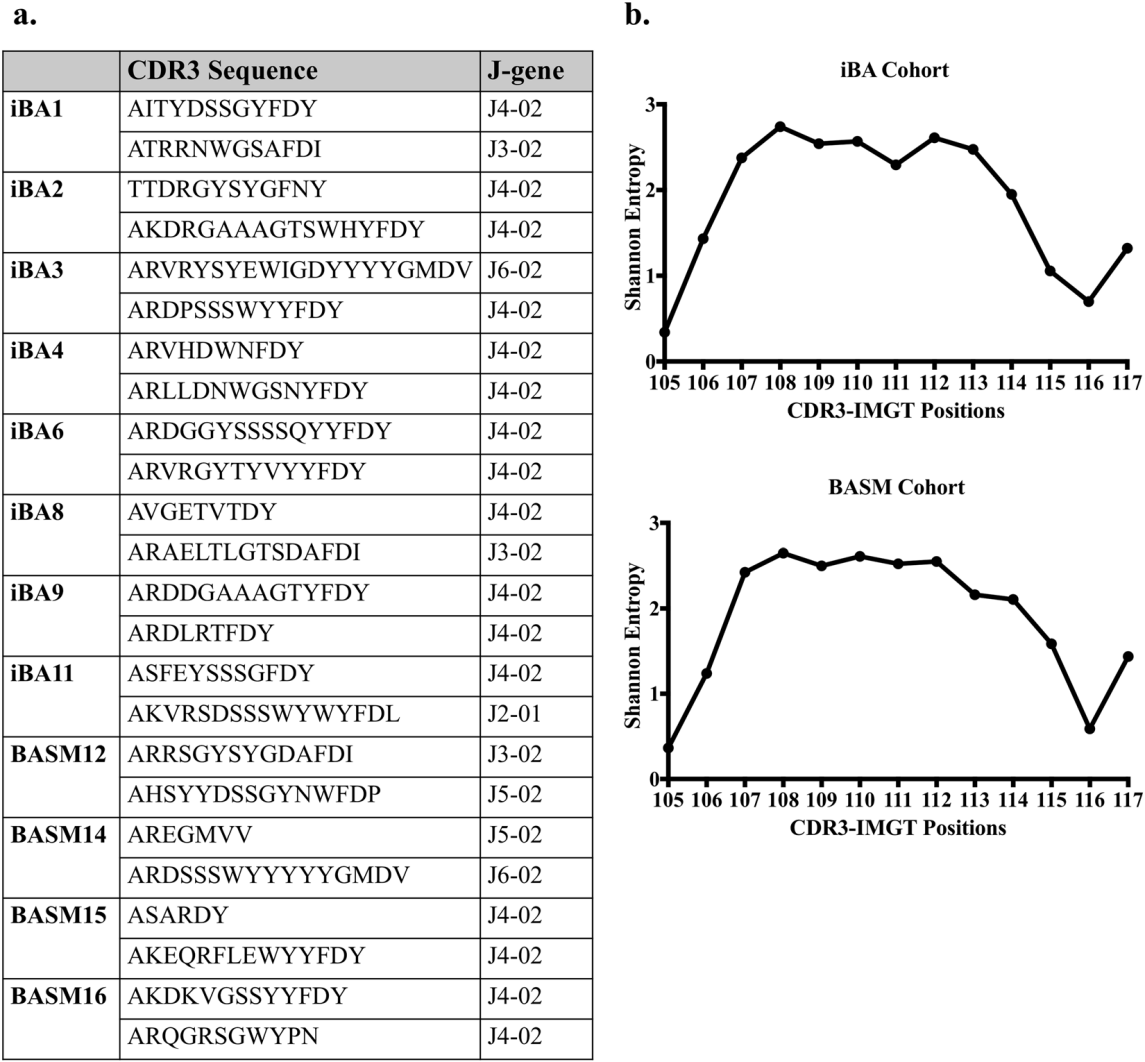
CDR3 sequence variation between biliary remnants. (**a**) There was little sequence homology between CDR3 sequences of BA cases. The J4-02 allele was present in the dominant CDR3 sequences of most biliary remnants. (**b**) Overall evaluation of amino acid variability by Shannon entropy demonstrated few conserved amino acid positions (Shannon entropy value <1) in the 14 amino acid CDR3 sequences of both the iBA (top right) and BASM (bottom right) disease groups.

We next compared the amino acid sequences of the CDR3 regions. Seventy-five percent of the biliary remnant Ig repertoires had the amino acid sequence FDY in at least one of the top two IgH CDR3 sequences (Fig. 5a). This is reflective of the dominant variable J-gene family J4 (allele J4-02) (Fig. 5a). There were few shared unique IgH CDR3 sequences within each disease group: there were 26 shared IgH CDR3s (0.06%) within the iBA group and 19 shared IgH CDR3s (0.07%) within the BASM group. Similarly, there were few shared CDR3 sequences between the disease groups with only 110 common IgH CDR3s (0.15%) between the iBA and BASM groups. We calculated the Shannon entropy for a CDR3 length of 14 amino acids to evaluate the amino acid variability at any given CDR3 position (Fig. 5b). Of the 14 amino acid positions within both the iBA and BASM groups, 2 positions were conserved in each group (Shannon entropy value <1). These results support a short shared sequence from the J4-02 allele in the majority of IgH CDR3 regions. Such sequence variation is not unexpected given individuals vaccinated with a known single antigen display significant sequence variability of the CDR3 region.

### Polyclonal immunoglobulin repertoire of local lymph nodes

**Figure 6:**
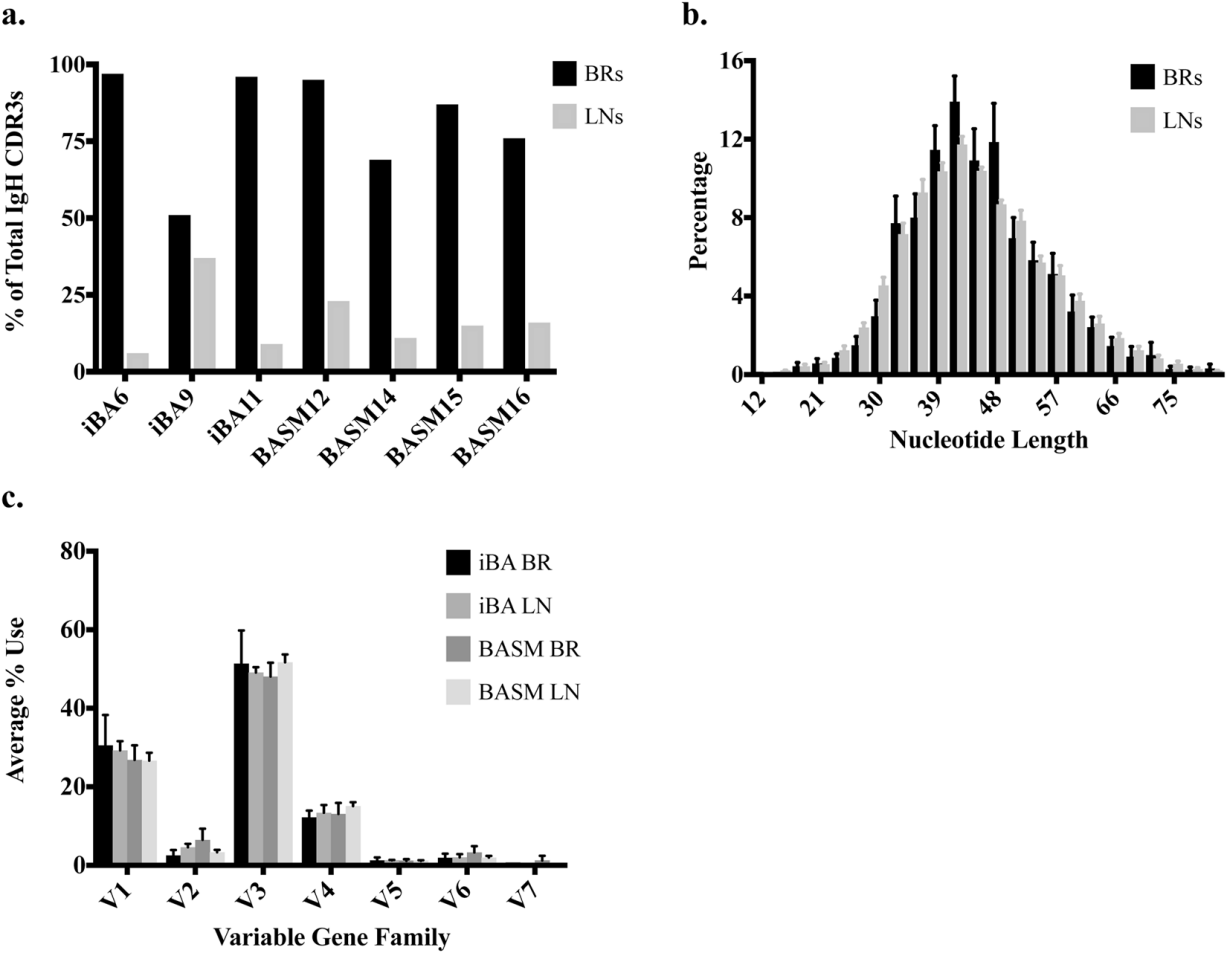
The immunoglobulin repertoire of local lymph nodes is more polyclonal. (**a**) The top 100 CDR3 sequences of the lymph nodes comprise a much smaller proportion of total sequences than their paired biliary remnants. D50 values for the biliary remnant and lymph node are 0.4 and 16.3 respectively. (**b**) Evaluation of paired biliary remnants and lymph nodes demonstrates a similar CDR3 length distribution. (**c**) Overall V-gene family use was similar between biliary remnants and lymph nodes for both the iBA and BASM disease groups.

Having demonstrated a highly clonal B cell population in the biliary remnants of iBA and BASM samples, we evaluated the Ig repertoire of the local lymph nodes from these same cases, the site where the B cell responses were likely generated. Not surprisingly, the local lymph nodes demonstrated a more polyclonal Ig repertoire than their paired biliary remnants (Fig. 6a). D50 values for the lymph nodes were higher than biliary remnants with an average lymph node D50 value of 12.1 (range 1.8–23) as compared to an average of 0.5 (range 0.1–0.9) for all biliary remnants (Table 2). Comparison of the number of clonotypes in the paired biliary remnants and lymph nodes for the different disease groups further supported this finding with clonotypes of iBA biliary remnants comprising 6.0% of total sequences as compared to 30% in the paired lymph nodes, and clonotypes of BASM biliary remnants comprising 6.8% of total sequences as compared to 32% in the paired lymph nodes.

Evaluation of the Ig phenotype of the local lymph nodes demonstrated a similar CDR3 length distribution and V-gene family use to the paired biliary remnants (Fig. 6b,c). Additionally, V-J gene associations were similar between the paired biliary remnants and lymph nodes of both disease groups. V1-69/J4 and V1-2/J4 were among the top 4 V-J gene associations for both the remnants and lymph nodes within the iBA group and V3-33/J4 and V3-30/J4 were among the top 4 V-J gene associations for the remnants and lymph nodes within the BASM group.

Evaluation of sequence homology between the paired biliary remnant and lymph node CDR3s demonstrated that on average, the biliary remnants shared 1.3% of unique IgH CDR3 sequences with the paired lymph node (range 0.7–2.5%). The top shared sequence for each pair was a CDR3 sequence with high frequency within the biliary remnant Ig repertoire. Within the 3 iBA pairs, the top shared sequence was the 12^th^, 14^th^, and 16^th^ most frequent IgH CDR3 sequence in the biliary remnant Ig repertoire. The top shared sequence in the 4 BASM pairs was the 1^st^, 2^nd^ (2 cases), and 4^th^ most frequent IgH CDR3 sequence in the biliary remnant Ig repertoire.

## Discussion

The etiology and immune mechanism of BA remains unknown and is limiting therapeutic advances for this disease. In the current study, we have defined the Ig repertoire of BA and provided evidence for an antigen-driven B cell immune response in diseased biliary remnants at diagnosis. We have shown that, similar to the T cell receptor repertoire^6^, the human BCR repertoire is highly clonal strongly supporting a role for antigen stimulation early in disease pathogenesis. Furthermore, we demonstrated that *both* BA subtypes, iBA and BASM, exhibit a highly clonal Ig repertoire. These data suggest that disease pathogenesis in these two distinct forms of BA may be more similar than previously thought. These results call into question the belief that BASM arises from a primary congenital insult rather than immune dysregulation as thought in iBA. Further research is needed to fully define possible similarities in the immune mechanism of these subtypes.

The prevailing theory for the development of autoreactive immune cell populations in BA includes exposure of a previously sequestered biliary protein, exposure to ‘self’ proteins that are altered either by a virus or environmental trigger, or molecular mimicry between a ‘self’ antigen and viral protein^2^. While our data have few shared CDR3 sequences between individual cases, this is consistent with known CDR3 sequence variability even in people with similar heritable variable gene use profiles^16^. In response to vaccination with a known antigen, sequence homology is limited to short amino acid motifs and dominant variable gene patterns rather than complete sequence homology between individuals^17–19^. While our data demonstrate similar CDR3 length distribution and similar dominant V-J gene associations between patients, analysis of the Ig phenotype is limited by sample size. Future studies including a larger cohort of BA infants may allow for discovery of a common disease-specific Ig phenotype in BA and identify new disease biomarkers.

Our study is limited by a lack of well-preserved non-diseased biliary remnant tissue to serve as controls. While we acknowledge this limitation for Ig repertoire analysis, we demonstrate that there are minimal B cells in choledochal cyst tissue as one would expect in healthy tissue without immune activation. Furthermore, Ig repertoire analysis of tissue without a significant B cell infiltrate would not provide a useful comparison due to significant differences in the abundance distribution of BCR sequences^20^. More importantly, we show that B cells in the biliary remnants are similar in abundance yet more clonal than local lymph nodes. This finding supports the conclusion that the Ig repertoire in biliary remnants of BA infants represents an antigen-driven disease phenotype in an otherwise non-inflammatory tissue. Additionally, while our sample size is small and we cannot exclude the possibility of a type II error, the consistency of our findings within each group strengthens the conclusions of this pilot study and supports future studies with a larger sample size. Another limitation of our study is the predominant white race of our patient population that may bias towards greater similarities in V-gene use if patients share heritable V-gene use patterns. Future work involving more BA infants will address this limitation.

While our data suggest that BA is an antigen-driven disease, further studies are needed to determine the exact role of B cells in disease pathogenesis. B cells are known to have various functions in autoimmune diseases and data suggests that dysregulated B cells clones play an early role in the development of autoimmune disease^21,22^. Our RT-PCR data of diseased tissue in these young BA infants demonstrates more CD19 than CD20 expressing cells in the biliary remnants and less CD20 expressing cells in the biliary remnants than local lymph nodes. As CD20 is downregulated on plasma cells, this finding suggests a different abundance of B cell subsets in the diseased tissue and supports a possible role for antibody-producing B cells in biliary remnants of both BA disease groups.

Based on the findings in the current study, future work will investigate the role of plasma cells in BA by synthesizing BA-specific monoclonal antibodies *in vitro* using the sequencing data derived from these oligoclonal B cell populations^11,23^. These synthetic BA-specific monoclonal antibodies will serve as powerful tools to identify candidate target antigen(s) in BA. Additional studies defining the Ig repertoire of diseased liver tissue of BA patients after Kasai portoenterostomy will provide insight into the role for B cells in ongoing liver injury with the ultimate goal to identify new therapeutic targets to slow or prevent disease progression.

## Materials and Methods

### Human BA tissue samples

This study was performed using formalin-fixed, paraffin-embedded tissue sections obtained from the pathology archives of Ann & Robert H. Lurie Children’s Hospital of Chicago. Biliary remnants, the diseased portion of the extrahepatic bile duct removed at surgery, and local lymph nodes were obtained from the tissue repository of the Childhood Liver Disease Research Network (ChiLDReN). These repository samples were obtained from BA patients less than 6 months of age at the time of diagnosis and surgical intervention with Kasai portoenterostomy (KPE). Biliary remnant samples were obtained from 11 infants with iBA and 5 infants with BASM. Local lymph nodes were obtained from 5 each of the iBA and BASM cases. Of the total 16 biliary remnant samples, 1 iBA sample did not yield sufficient RNA for deep sequencing analysis and 3 samples (2 iBA and 1 BASM) yielded deep sequencing results of poor quality and were excluded from analysis. This provided a total of 8 iBA biliary remnants of which 3 had paired lymph nodes and 4 BASM biliary remnants each with paired lymph nodes. Laboratory data were collected at baseline for each patient and reviewed retrospectively. Approval for conducting the study was obtained from the Institutional Review Board of Lurie Children’s Hospital of Chicago. All methods were carried out in accordance with the Institutional Review Board’s guidelines and regulations. Enrollment in the ChiLDReN study required informed consent, including laboratory studies and the use of tissue samples in research. Informed consent was obtained from a parent and/or legal guardian for study participants under the age of 18 years. ChiLDReN approved their use in the current study as an ancillary study.

### Quantitation of B cells in biliary remnants of iBA and BASM cases: immunohistochemistry and RT-PCR

Immunohistochemistry was performed on biliary remnants removed from infants with BA at the time of KPE. Formalin-fixed and paraffin-embedded tissue sections were stained with the antibody to the B cell lineage marker Pax5 (Clone SP34, Ventana, Tucson, Arizona) for 32 minutes at 37 °C. For comparison of B cell abundance in non-BA biliary tissue, formalin-fixed and paraffin-embedded tissue sections from pediatric choledochal cyst remnants were stained with Pax5 (Clone 24, BD Biosciences, San Jose, California) for 15 minutes at room temperature. Quantitative analysis for Pax5 was performed using Nikon Elements Advanced Imaging and Analysis software. Analysis was completed on the full slides for 4 iBA (2 cases removed due to inaccurate quantification from tissue processing), 4 BASM, and 4 choledochal cyst cases. Additional quantitative analysis was performed on the high-powered field (40x magnification) with the densest B cells for 6 iBA, 4 BASM, and 4 choledochal cyst cases. Unpaired t-test was used to calculate statistically significant differences between groups.

For quantification of B cells by RT-PCR, total RNA isolated from iBA and BASM biliary remnants and lymph nodes was reverse transcribed into cDNA using the iScript Advanced cDNA synthesis kit (Bio-Rad, Hercules, CA). Each cDNA sample was pre-amplified with SsoAdvanced PreAmp supermix (Bio-Rad, Hercules, CA), which is optimized for unbiased target-specific pre-amplification of target genes using the specific primer pool for CD19, Pax5, CD20, and β-actin genes. Quantitative real-time PCR (qPCR) was performed with the specific primers and fold change (2^ΔCt^) of our samples was calculated relative to a calibrator sample (liver tissue from a BA patient) and normalized to β-actin expression. Differences in the average relative quantification of B cell markers between groups was compared using the unpaired t-test.

### Analysis of the immunoglobulin repertoire by multiplex PCR technology

RNA was isolated from iBA (8 biliary remnants and 5 lymph nodes) and BASM (4 biliary remnants and 5 lymph nodes) samples. Automated multiplex PCR was performed on the immunoglobulin heavy (IgH) and light (IgL) chain cDNA to yield variable sequences for unique IgH and IgL chains that comprise the Ig repertoire (iRepertoire, Inc., Huntsville, Alabama). Output generated 300,000–400,000 reads per library. Analysis of clonality was limited to IgH sequences in the current study as they more accurately reflect clonal selection in an antigen-driven disease. Within the IgH variable region, the CDR3 sequence is most closely associated with antigen-specificity and is the focus of Ig repertoire analysis.

### Statistical analysis of antibody sequences

A limit of 50,000 FASTA files per sample were uploaded to IMGT/HighV-Quest (IMGT®, the international ImMunoGeneTics information system®, http://www.imgt.org; founder and director Marie-Paule Lefranc, Montpellier, France)^24–26^ for standardized analysis of the Ig nucleotide sequence. All iBA and BASM output files from IMGT/HighV-Quest were grouped together and uploaded to IMGT/StatClonotype for pairwise comparison between the two disease groups^27,28^. R software (version 3.3.3) was used to determine the number of shared CDR3 sequences within and between disease groups.

Clonal expansion of the Ig repertoire of each sample was evaluated by the CDR3 sequence variability. Statistical output from BCR sequencing by iRepertoire generates the D50 value as a single number to reflect a sample’s diversity. The D50 value represents the percent of dominant and unique B cell clones that account for the cumulative 50% of the total CDR3s in the sample. A D50 value of 50 would thus reflect a completely heterogeneous sample with each sequence contributing equally as one would expect in a naïve B cell population whereas a D50 of 0 would reflect a monoclonal sample in which the sequences were derived from a single B cell clone. Assessment of sample clonality was also determined by calculation of the percent contribution of the top 100 most prevalent CDR3s, such that in a highly clonal repertoire the top 100 CDR3s would comprise the majority of total CDR3s. Further data analysis by IMGT/StatClonotype yielded the number of clonotypes per disease group where IMGT clonotypes are defined as groups with a unique V-(D)-J rearrangement and a unique CDR3-IMGT amino acid junction sequence using conserved CDR3-IMGT anchors^27^.

The Ig phenotype of iBA and BASM biliary remnants and lymph nodes was evaluated by assessment of the average CDR3 sequence nucleotide length for each disease group. For additional analysis of the Ig phenotype, variation in the average percent use of variable V-gene families and V-gene alleles was calculated for all samples within each disease group and the standard error of the mean was reported. Variable V-gene use was compared between disease groups for biliary remnants, and between paired biliary remnants and lymph nodes. Association between variable V-gene and J-gene alleles within disease groups was evaluated using IMGT/StatClonotype.

The total number of shared CDR3 sequences within a disease group and between disease groups was determined using R software (version 3.3.3). The percent shared sequences of total CDR3 sequences was calculated using total number of unique CDR3 sequences for the entire disease group. Shannon entropy for each disease group was calculated using IMGT/StatClonotype to provide additional evaluation of the variation of amino acid identity at a given CDR3 position.

## Data Availability

Sequencing data is publicly available on GenBank. The additional datasets generated during and/or analyzed during the current study are available from the corresponding author on reasonable request.
